# Susceptibility profile of *bla*_OXA-23_ and metallo-β-lactamases co-harbouring isolates of carbapenem resistant *Acinetobacter baumannii* (CRAB) against standard drugs and combinations

**DOI:** 10.3389/fcimb.2022.1068840

**Published:** 2023-01-06

**Authors:** Swati Sharma, Tuhina Banerjee, Ghanshyam Yadav, Ashok Kumar

**Affiliations:** ^1^ Department of Microbiology, Institute of Medical Sciences, Banaras Hindu University, Varanasi, India; ^2^ Department of Anaesthesiology, Institute of Medical Sciences, Banaras Hindu University, Varanasi, India; ^3^ Department of Pediatrics, Institute of Medical Sciences, Banaras Hindu University, Varanasi, India

**Keywords:** *bla*_NDM_, minocycline, meropenem, synergy, endemic

## Abstract

**Background:**

The rapid emergence of carbapenem resistant *Acinetobacter baumannii* (CRAB) has resulted in an alarming situation worldwide. Realizing the dearth of literature on susceptibility of CRAB in genetic context in the developing region, this study was performed to determine the susceptibility profile against standard drugs/combinations and the association of *in-vitro* drug synergy with the prevalent molecular determinants.

**Methods and findings:**

A total of 356 clinical isolates of *A. baumannii* were studied. Confirmation of the isolates was done by amplifying *recA* and ITS region genes. Susceptibility against standard drugs was tested by Kirby Bauer disc diffusion. Minimum inhibitory concentration (MIC), MIC_50_ and MIC_90_ values against imipenem, meropenem, doripenem, ampicillin/sulbactam, minocycline, amikacin, polymyxin B, colistin and tigecycline was tested as per guidelines. Genes encoding enzymes classes A (*bla*_GES_, *bla*_IMI/NMC-A_, *bla*_SME_, *bla*_KPC_), B (*bla*_IMP_, *bla*_VIM_, *bla*_NDM_) and D (*bla*_OXA-51,_ *bla*_OXA-23_ and *bla*_OXA-58_) were detected by multiplex polymerase chain reaction. Synergy against meropenem-sulbactam and meropenem-colistin combinations was done by checkerboard MIC method. Correlation of drug synergy and carbapenemase encoding genes was statistically analyzed.

**Results:**

Of the total, resistance above 90% was noted against gentamicin, ciprofloxacin, levofloxacin, ceftazidime, cefepime, ceftriaxone, cotrimoxazole and piperacillin/tazobactam. By MIC, resistance rates from highest to lowest was seen against imipenem 89.04% (n=317), amikacin 80.33% (n=286), meropenem 79.49% (n=283), doripenem 77.80% (n=277), ampicillin/sulbactam 71.62% (n=255), tigecycline 55.61% (n=198), minocycline 14.04% (n=50), polymyxin B 10.11% (n=36), and colistin 2.52% (n=9). CRAB was 317 (89.04%), 81.46% (n=290) were multidrug resistant and 13.48% (n=48) were extensively drug resistant. All the CRAB isolates harboured *bla*_OXA-51_ gene (100%) and 94% (n=298) *bla*_OXA-23_ gene. The *bla*_IMP_ gene was most prevalent 70.03% (n=222) followed by *bla*_NDM,_ 59.62% (n=189). Majority (87.69%, 278) were co-producers of classes D and B carbapenemases, *bla*_OXA-23_ with *bla*_IMP_ and *bla*_NDM_ being the commonest. Synergy with meropenem-sulbactam and meropenem-colistin was 47% and 57% respectively. Reduced synergy (*p*= <0.0001) was noted for those harbouring *bla*_OXA-51_+bla_OXA-23_with *bla*_NDM_ gene alone or co-producers.

**Conclusion:**

Presence of *bla*_NDM_ gene was a significant cause of synergy loss in meropenem-sulbactam and meropenem-colistin. In *bla*_NDM_ endemic regions, tigecycline, minocycline and polymyxins could be viable options against CRAB isolates with more than one carbapenemase encoding genes.

## 1 Introduction

The rapid emergence and widespread dissemination of carbapenem resistance in Gram negative bacilli has posed real challenges in the management of infection caused by them. In this regard, the emergence of carbapenem resistant *Acinetobacter baumannii* (CRAB) has been very significant not only because of the carbapenem resistance acquired by these organisms but also due to the fact that acquisition of this resistance has made the otherwise ‘insignificant colonizers’, a potential pathogen. The impact has been so severe that both the World Health Organization (WHO) in its global priority pathogen list and India in its Indian Pathogen Priority List has labelled CRAB as ‘critical priority pathogen’ for further research (World Health Organization Press [WHO], 2017; WHO and DBT, Indian Priority Pathogen List [IPPL], 2021). According to Global Antimicrobial Resistance Surveillance System (GLASS) report 2019, 68-82% percentage of CRAB isolates have been reported from Saudi Arabia, Egypt, South Africa, Argentina, Brazil, Iran, Pakistan, and Italy (GLASS, 2019). Moreover, the data from Central Asian and Eastern European surveillance of Antimicrobial Resistance (CAESAR) 2019, showed 80%-91% of CRAB isolates in Russia, Ukraine and Belarus (CAESAR, 2019). Similarly, the China Antimicrobial Surveillance network (CHINET) 2017 reported 82% CRAB isolates (CHINET, 2017) (OneHealth Trust).

Carbapenem group of drugs are the last resort therapeutic option in many low resource settings especially, in developing regions. However, as has been the case in India or for that matter most of the developing countries, the broad-spectrum property of this important group of drugs has encouraged excessive inappropriate use in form of over-the-counter scale or those without valid prescriptions (Laxminarayan and Chaudhury, 2016). Among the different enzymatic and non-enzymatic mechanisms of carbapenem resistance in CRAB like Ambler classes A/B/D, porin channels, and efflux pumps, Ambler class B metallo-beta lactamases (MBLs) like *bla*_NDM-1_, has been reported as most worrisome (López et al., 2019). In addition to this, in Indian scenario, CRAB is very different from other parts of the world. Not only the molecular determinants of carbapenem resistance varies, the combination of resistance genes and availability of alternative therapeutic options also pose huge challenge in deciding for their appropriate management (Bartal et al., 2022). Several studies, though limited by heterogeneity in methods and sample size, have reported synergistic effect of antibiotics combinations (Ayoub Moubareck and Hammoudi Halat, 2020; Mohd Sazlly Lim et al., 2021). Despite there is lack of epidemiological data and experimental studies on susceptibility to alternative options in Indian context which indirectly promotes empirical use of antibiotics and hence emergence of carbapenem resistant organisms.

We have previously identified and studied the endemicity of CRAB in the intensive care unit (ICU) of the present study center against a background of high empirical carbapenem use (Banerjee et al., 2018). We have also studied sustained outbreak of CRAB wherein, it was shown that intense carbapenem use within the ICU facilitated the persistence of the CRAB isolates in the hospital environment causing repeated outbreaks (Sharma et al., 2021a). We then studied colistin resistance in CRAB isolates wherein all the resistant isolates were reported in patients with prior carbapenem therapy (Sharma et al., 2021b). To meet the heavy empirical carbapenem use we also tried to restrict the empirical therapy by detecting biomass through a low-cost hand-held microscope (Foldscope) (Sharma et al., 2022). However, even though the challenge of CRAB infection was elucidated through this series of related studies, no consensus could be reached on the therapeutic options of these resistance strains. Realizing the scarcity of data in Indian context, the present study was conducted to determine the susceptibility profile of CRAB against available standard drugs and their combinations and to determine association of *in-vitro* drug synergy with the widely prevalent molecular determinants of carbapenem resistance. To the best of our knowledge, this study provides the data on drug synergy and epidemiology on the largest number of CRAB isolates.

## 2 Materials and methods

### 2.1 Study site

This prospective cross-sectional study was conducted in the Department of Microbiology, Institute of Medical Sciences, Banaras Hindu University, and the associated 2000 bedded tertiary care hospital, Varanasi. The work was approved by Institute ethical committee (Dean/2017/EC/186) and prior to sample collection an informed consent was taken from each subject or their guardian.

### 2.2 Bacterial isolates

Isolates of *A. baumannii* from different clinical specimens were included in the study. The isolates were collected from various samples from the patients admitted to different wards and ICUs of the hospital over a period of 15 months (January 2018-March 2019). The sample size was calculated by the formula ‘n=Z^2^pq/d^2^’ (n=minimum sample size, Z= standard score based on given confidence level, p=prevalence rate, q=1-p, d= standard error), considering the previous prevalence data of *A. baumannii* in the study center (Banerjee et al., 2018; Sharma et al., 2022). More than required isolates were included to increase the power of study and to eliminate any bias. The detailed demographic data of the patients were also noted.

### 2.3 Inclusion and exclusion criteria

Only those isolates were considered which were collected from patients with clinical suspicion of infections like pneumonia, skin and soft tissue infection, sepsis, and urinary tract infection. Only the first isolate from the samples were included. *A. baumannii* isolated from mixed infections and those suggesting colonization were excluded.

### 2.4 Isolation and identification

All the isolates were phenotypically characterized by standard microbiological methods as culture on MacConkey agar and Leeds *Acinetobacter* agar base media (HiMedia Laboratories Pvt Ltd, India), Gram staining and biochemical reactions. The molecular identification as *A. baumannii* was done by multiplex PCR, targeting *recA* gene and species specific ITS-region gene (Fallon and Young, 1996; Chen et al., 2014).

### 2.5 Antimicrobial susceptibility testing

#### 2.5.1 Disc diffusion method

Susceptibility towards gentamicin (10 µg), ciprofloxacin (5 µg), levofloxacin (5 µg), ceftazidime (30 µg), cefepime (30 µg) ceftriaxone (30 µg), cotrimoxazole (1.25/23.75 µg), piperacillin/tazobactam (100/10 µg), ampicillin/sulbactam (10/10 µg), imipenem (10 µg), meropenem (10 µg) and amikacin (HiMedia Laboratories Pvt. Ltd, India) was tested by Kirby Bauer disc diffusion method.

#### 2.5.2 Determination of minimum inhibitory concentration (MIC) against selected drugs

MIC for imipenem, meropenem, doripenem, ampicillin, sulbactam, tigecycline, colistin (Sigma-Aldrich Chemicals Pvt. Ltd, India), polymyxin B (Bharat serums & vaccines Ltd, India), amikacin (Aristo Pharmaceuticals Ltd. India) and minocycline (Gufic Biosciences Ltd, India) was performed by agar dilution or broth microbroth dilution methods as per recommendation by Clinical Laboratory Standards Institute (CLSI) guidelines (CLSI, 2020). The bacterial inoculum was prepared by inoculating, 2-3 pure isolated colonies from overnight growth into Luria Bertani (LB) broth medium (HiMedia Laboratories Pvt Ltd, India) and incubated at 37°C with constant shaking at 180 rpm for 2 hours. The turbidity was adjusted according to 0.5 McFarland standards and 0.01 mL suspension was used as inoculum. The test was performed in cation-adjusted Mueller Hinton broth and agar medium (HiMedia Laboratories Pvt Ltd, India). The drug potency was calculated as described elsewhere and antibiotic stock solution was prepared by dissolving antibiotic powders into appropriate solvent (Biswas and Rather, 2019). *Escherichia coli* ATCC*^®^* 25922, *Pseudomonas aeruginosa* ATCC*^®^ 27853* and *Acinetobacter baumannii* ATCC*^®^ 19606* were used as quality controls. The results were interpreted according to CLSI guidelines 2020 (CLSI, 2020). For tigecycline, isolates with ≥4 µg/ml MIC were considered as resistant isolates (Marchaim et al., 2014).

### 2.6 Determination of MIC_50_ and MIC_90_

For each tested antibiotic the MIC_50_ and MIC_90_ value was calculated. The MIC_50_ is equivalent to median MIC value and calculated as n x 0.5 (n=no. of test isolates). The MIC_90_ is the 90^th^ percentile of the MIC value and calculated as n x 0.9, if the resulting number wasn’t an integer, therefore the subsequent integer next to the respective value represented the MIC_90_ (Biswas and Rather, 2019).

### 2.7 Definitions and determination of multiple antibiotic resistance (MAR) index

CRAB was defined as, an isolate resistant to anyone carbapenem (imipenem or meropenem). Multi-drug resistant *A. baumannii* (MDRAb) and extensively-drug resistant *A. baumannii* (XDRAb) was defined as an isolate showing non-susceptibility to at least 1 agent in ≥3 antimicrobial categories and at least 1 agent in all but <2 or fewer antimicrobial categories, respectively including penicillins, ß-lactam combination agents, cephems, carbapenems, lipopeptides, aminoglycoside, tetracyclines, fluoroquinolones, and folate pathway antagonists (Magiorakos et al., 2012). The result of disc diffusion method was used for the above classification except for lipopeptides and tetracyclines which were not tested by disc diffusion method.

The MAR index was determined by using the formula MAR = a/b, where ‘a’ is the number of antibiotics to which the test isolate showed resistance and ‘b’ is the total number of antibiotics to which the test isolate was exposed. Values >0.2 MAR index represents high risk source of contamination is where antibiotics are frequently used (Sandhu et al., 2016).

### 2.8 Detection of carbapenemase encoding genes

The phenotypically carbapenem resistant isolates as detected by their MICs were subjected to genotypic characterization of carbapenemases encoding genes. Four different multiplex PCR was performed for detection of class A (*bla*_GES_, *bla*_IMI/NMC-A_, *bla*_SME_, *bla*_KPC_), class B (*bla*_IMP_, *bla*_VIM_, *bla*_NDM_) and class D (*bla*_OXA-51,_ *bla*_OXA-23_ and *bla*_OXA-58_) genes. Each single reaction mixture (25 µL) contained 2.5 µL Taq DNA buffer, 2 µL of dNTP, and 1 µL of each primer (10 picomole; Eurofins Scientific India Pvt. Ltd.), 0.3 µL of Taq DNA polymerase (Genei Laboratories Pvt. Ltd., India). To maintain volume, 5 µL of template DNA (100 ng/mL) and nuclease free water was added. The reactions were run under the following conditions: For *bla*_GES_, *bla*_IMI/NMC-A_, *bla*_SME_, and *bla*_KPC_ genes, initial denaturation at 94°C for 5 min, 25 cycles at 94°C for 30 sec, 50°C for 30 sec, 72°C for 60 sec, and final extension at 72°C for 7 min (Hong et al., 2012). For *bla*_IMP_, *bla*_VIM_, and *bla*_NDM_ genes, initial denaturation at 94°C for 10 min, 36 cycles at 94°C for 30 sec, 52°C for 40 sec, 72°C for 50 sec, and final extension at 72°C for 5 min (Poirel et al., 2011). For *bla*_OXA-51,_ and *bla*_OXA-23_ genes, initial denaturation at 94°C for 3 min, 35 cycles at 94°C for 45 sec, 57°C for 45 sec, 72°C for 60 sec, and final extension at 72°C for 5 min (Turton et al., 2006). For *bla*_OXA-58_ gene, initial denaturation at 94°C for 5 min, 30 cycles at 94°C for 25 sec, 52°C for 40 sec, 72°C for 50 sec, and final extension at 72°C for 6 min (Woodford et al., 2006). The primer pairs used in the study have been shown in **Table 1**.

**Table 1:** Primer sequences used in the study.

S.No.	Primer pairs	Sequence (5’-3’)	Target	Base-pair	Ref.
1	P-rA1 P-rA2	CCTGAATCTTCTGGTAAAAC GTTTCTGGGCTGCCAAACATTAC	*recA*	425	Chen et al., 2014
2	P-Ab-ITSF P-Ab-ITSB	CATTATCACGGTAATTAGTG AGAGCACTGTGCACTTAAG	ITS	208	
3	GES-F GES-MR	GCTTCATTCACGCACTATT CGATGCTAGAAACCGCTC	*bla*_GES1-9, 11-20_	323	Hong et al., 2012
4	IMI(NMC)-F1 IMI(NMC)-R1	TGCGGTCGATTGGAGATAAA CGATTCTTGAAGCTTCTGCG	*bla*_IMI13_ and *bla*_NMC-A_	399	
5	SME-F1 SME-R1	ACTTTGATGGGAGGATTGGC ACGAATTCGAGCATCACCAG	*bla*_SME1-3_	551	
6	KPCF2 KPCFR	GTATCGCCGTCTAGTTCTGC GGTCGTGTTTCCCTTTAGCC	*bla*_KPC2-13_	638	
7	IMP-F IMP-R	GGAATAGAGTGGCTTAAYTCTC GGTTTAAYAAAACAACCACC	*bla*_IMP_	232	Poirel et al., 2011
8	VIM-F VIM-R	GATGGTGTTTGGTCGCATA CGAATGCGCAGCACCAG	*bla*_VIM_	390	
9	NDM-F NDM-R	GGTTTGGCGATCTGGTTTTC CGGAATGGCTCATCACGATC	*bla*_NDM_	621	
10	OXA-23-like F OXA-23-like R	GATCGGATTGGAGAACCAGA ATTTCTGACCGCATTTCCAT	*bla*_OXA-23_	501	Turton et al., 2006
11	OXA-51-like F OXA-51-like R	TAATGCTTTATCGGCCTTG TGGATTGCACTTCATCTTGG	*bla*_OXA-51_	353	
12	OXA-58-like F OXA-58-like R	AAGTATTGGGGCTTGTGCTG CCCCTCTGCGCTCTACATAC	*bla*_OXA-58_	599	Woodford et al., 2006
13	Rep1 Rep2	IIIGCGCCGICATCAGGC ACGTCTTATCAGGCCTAC	–	–	Fitzpatrick et al., 2016

### 2.9 Drug combination testing

For 100 selected CRAB isolates with different genetic profile, synergy testing was performed in 96-well microtiter plate by checkerboard MIC method. The selection of antibiotics for synergy testing was done after reviewing the antibiogram of the tertiary care center and literature on potentially potent antibiotic combinations for CRAB isolates (Laishram et al., 2017; Banerjee et al., 2018; Ayoub Moubareck and Hammoudi Halat, 2020). The synergy was investigated for combination of meropenem with sulbactam and meropenem with colistin. The concentration used for meropenem-sulbactam combination ranged from 2-256 µg/ml for meropenem and 2-128 µg/ml for sulbactam. For meropenem-colistin combination, the concentration for meropenem used was same as above and for colistin the concentration ranged from 0.5-2 µg/ml. Single agent MIC was also determined during the checkerboard assay. The fractional inhibitory concentration index (FICI) was calculated and interpreted as described earlier (Biswas and Rather, 2019).

### 2.10 Molecular typing

The clonal relationship of 100 CRAB isolates included for combination testing was studied by repetitive extragenic palindromic polymerase chain reaction (Rep-PCR) as described earlier (Fitzpatrick et al., 2016). The primer pair Rep1and Rep2 was used for the amplification. The reaction was run under the following condition, initial denaturation at 94°C for 3 min, 30 cycles at 94°C for 60 sec, 40°C for 60 sec, 65°C for 8 min, and final extension at 72°C for 16 min. Each single reaction mixture (25 µl) contained 2.5 µl Taq DNA buffer, 2 µl of dNTP, and 2µl of each primer (10 picomole; Eurofins Scientific, India), 0.3 µl of Taq DNA polymerase (Genei, Bangalore, India). To maintain volume, 5 µl of template DNA (100 ng/mL) and nuclease free water was added. The amplified PCR products were run on 1.8% agarose gel electrophoresis (BioRad Laboratories India Pvt. Ltd, India). Further the isolates showing similar band pattern were considered as one Rep cluster while isolates with inconsistent bands were grouped into different Rep cluster based on the dendrogram.

### 2.11 Statistical analysis

Fisher’s exact test was employed with the help of MedCalc^®^ statistical software version 19.6.3.0., to compare the synergistic effect of drug combination with the phenotypic resistance profile and molecular determinants of carbapenem resistance in the CRAB isolates respectively.

## 3 Results

### 3.1 Bacterial isolates

**Figure 1:**
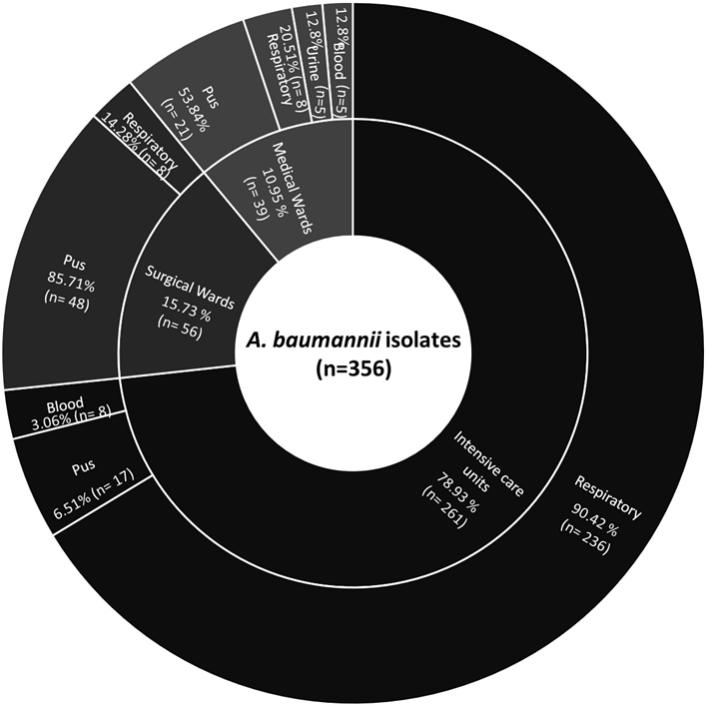
Sunburst chart showing distribution of *A. baumannii* isolates among different departments and clinical specimen.

A total of 356 A*. baumannii* isolates confirmed by *recA* and *ITS* gene amplification were studied, among which majority were from the ICU 71.91% (n=256) followed by surgical wards 14.60% (n=52), and medical wards 13.48% (n=48). The most frequent site of infection was the lower respiratory tract. The demographic details of the patients showed, 71.1% (n=253) were males and 28.9% (n=103) were female with the mean age of 35.6 and 40.4 years respectively. The distribution of isolates among different clinical specimens and department has been shown in **Figure 1**.

### 3.2 Susceptibility

#### 3.2.1 Disc diffusion method

Among the 356 isolates, resistance was noted against gentamicin 93.25% (n=332), ciprofloxacin 96.06% (n=342), levofloxacin 92.13% (n=328), ceftazidime 94.66% (n=337), cefepime 96.34% (n=343), ceftriaxone 97.75% (n=348), cotrimoxazole 91.85% (n= 327), piperacillin/tazobactam 93.25% (n=332), ampicillin/sulbactam 76.93% (n=274), imipenem 92.41% (n=329), meropenem 87.35% (n=311) and amikacin 85.67% (n=305) by disc diffusion assay.

#### 3.2.2 Determination of minimum inhibitory concentration (MIC) against selected drugs

By MIC, highest resistance was seen against imipenem 89.04% (n=317) followed by amikacin 80.33% (n=286), meropenem 79.49% (n=283), doripenem 77.80% (n=277), ampicillin/sulbactam 71.62% (n=255), tigecycline 55.61% (n=198), minocycline 14.04% (n=50), polymyxin B 10.11% (n=36), and colistin 2.52% (n=9). The MIC results were considered for those drugs that were tested by both the methods, in case of discrepancy. The total number of isolates that were classified as CRAB were 317 (89.04%). Among 356 isolates, 81.46% (n=290) were reported as MDRAb, and 13.48% (n=48) as XDRAb. The exact MIC range, MIC_50_ and MIC_90_ values for each antimicrobial agent has been summarized in **Table 2**.

**Table 2:** Minimum inhibitory concentration range, MIC_50_ and MIC_90_ values of *A.baumannii* isolates.

Antimicrobial Agents	MIC range (µg/ml)	MIC_50_	MIC_90_
Ampicillin/sulbactam	0.5 – >128	64	128
Imipenem	0.5 – >256	128	256
Meropenem	0.5 – >128	64	>128
Doripenem	0.5 – >128	32	128
Polymyxin B	0.5 – 64	1	4
Colistin	0.5 – 64	1	2
Amikacin	4 – >512	128	512
Minocycline	0.5 – 64	4	16
Tigecycline	0.5 – 128	32	64

MAR index revealed 36 drug resistance patterns against 9 antimicrobial agents and >2 MAR index in 54.77% isolates (**Supplementary Table 1**). All of them were isolated from the ICU.

### 3.3 Carbapenemase encoding determinants

**Figure 2:**
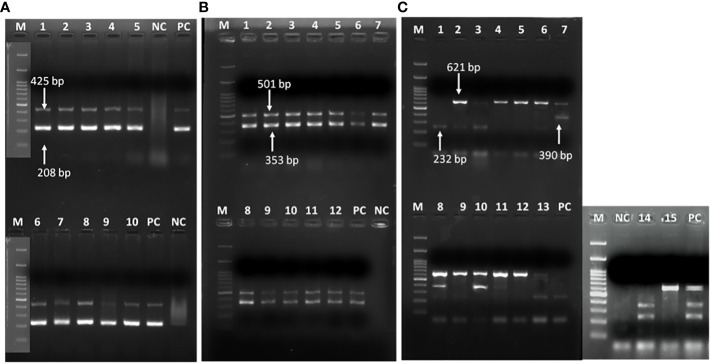
Representative gel image showing **(A)** *recA* and ITS genes in *A baumannii*; **(B)** *bla*_OXA-51_ and *bla*_OXA-23_ genes; **(C)** multiple class B carbapenemases. **(A)** Lane M: Marker 100 bp; Lane 1-5,6-10: *recA* (425bp) & *ITS* gene (208bp); NC: negative control PCR-grade water; PC: positive control *A. baumannii* ATCC 19606. **(B)** class D carbapenemase genes; Lane M: Marker 100 bp; Lane 1-12: *bla*_OXA-51_ (353 bp) & Lane 1-12: *bla*_OXA-23_ (501 bp); NC: negative control PCR-grade water; PC: previously confirmed & published isolate positive for *bla*_OXA-51_ & *bla*_OXA-23_ genes **(C)** class B carbapenamse genes; Lane M: Marker 100 bp; Lane 1-3,6,8, 13, 14: *bla*_IMP_ (232 bp), Lane 7-8,10-11, 14: *bla*_IMP_ (390 bp) & Lane 2-13, 15: *bla*_NDM_ (621 bp); PC: previously confirmed & published isolate positive for *bla*_IMP_, *bla*_VIM_ and *bla*_NDM_ genes.

The genotypic characterization of 317 CRAB isolates showed that all were carrying *bla*_OXA-51_ gene (100%) and 94% (n=298) of the isolates were harbouring *bla*_OXA-23_ gene. Among class B carbapenemases, *bla*_IM_ *_P_* gene was most prevalent 70.03% (n=222) in the CRAB isolates followed by *bla*_NDM,_ 59.62% (n=189) and *bla*_VIM_, 31.23% (n=99) genes. Majority of isolates, 87.69% (n=278) were co-producers of class D and class B carbapenemases in multiple combinations (**Figure 2**). The most common combination was *bla*_OXA-23_ with *bla*_IMP_ and *bla*_NDM_ gene. None of the isolate was found positive for class A carbapenemases genes and *bla*_OXA-58_. The association between phenotypic carbapenem resistance profile and genotypic resistance profile of CRAB isolates has been shown in **Table 3**.

**Table 3:** The comparison of the phenotypic carbapenem resistance profile and genotypic resistance profile of CRAB isolates.

Phenotypic resistance profile	n	Genotypic profile	N
IPM/MEM/DOR	272	*bla*_OXA-51_+*bla*_OXA-23_+*bla*_IMP_+*bla*_NDM_+*bla*_VIM_	28
		*bla*_OXA-51_+*bla*_OXA-23_+*bla*_IMP_+*bla*_NDM_	114
		*bla*_OXA-51_+*bla*_OXA-23_+*bla*_IMP+_ *bla*_VIM_	62
		*bla*_OXA-51_+*bla*_OXA-23_+*bla*_NDM_	46
		*bla*_OXA-51_+*bla*_OXA-23_+*bla*_IMP_	13
		*bla*_OXA-51_+*bla*_OXA-23_+*bla*_VIM_	9
IPM/MEM	11	*bla*_OXA-51_+*bla*_OXA-23_ +*bla*_NDM_ *bla*_OXA-51_+*bla*_OXA-23_	1 10
IPM/DOR	5	*bla*_OXA-51_+*bla*_OXA-23_+*bla*_IMP_	5
IPM	29	*bla*_OXA-51_+*bla*_OXA-23_	10
		*bla*_OXA-51_	19

IPM, imipenem; MEM, meropenem; DOR, doripenem.

### 3.4 Drug combination testing

The reduction in MIC range, MIC_50_ and MIC_90_ was noted against the antibiotics in combination as compared to antibiotics as single agent (**Table 4**).The MIC_50_ and MIC_90_ of meropenem and sulbactam was reduced four-fold when tested in combination. The combination of meropenem-sulbactam was synergistic against 47% CRAB isolates and indifference against 53% CRAB isolates. When meropenem was combined with colistin, eight-fold and four-fold reduction in MIC_50_ and MIC_90_ of meropenem and colistin was noted respectively. The meropenem-colistin combination showed 57% synergy and 43% indifference against CRAB isolates. None of the combination showed antagonistic effect (**Supplementary Table 2**).

**Table 4:** Summarized results for drug combinations tested by checkerboard method against 100 CRAB isolates.

	Single agent MIC (µg/ml)			MEM+SUL Combination MIC (µg/ml)		MEM+SUL ΣFICI	Interpretation	MEM+COL Combination MIC (µg/ml)		MEM+COL ΣFICI	Interpretation
	MEM	SUL	COL	MEM	SUL			MEM	COL		
**Range**	8-256	16-128	0.5-2	2-128	1-64	0.31-1.5	47% synergy 53% indifference	0.5-32	0.25-2	0.13-1.12	57% synergy 43% indifference
**MIC_50_**	128	64	1	32	16	–		16	0.5	–	
**MIC_90_**	256	128	2	64	32	–		32	1	–	

MIC, minimum inhibitory concentration; MEM, meropenem; SUL, sulbactam; COL, colistin; FICI, fractional inhibitory concentration index.

The synergistic effect of drug combinations was compared with the molecular mechanism of carbapenem resistance in CRAB isolates (**Table 5**). For both the combinations meropenem-sulbactam and meropenem-colistin 90-100% synergy was observed for isolates carrying *bla*_OXA-51_+*bla*_OXA-_ *_23_* and *bla*_OXA-51_+*bla*_OXA-23_with *bla*_VIM_ or *bla*_IMP_ genes. However, significantly lower synergy (*p*= <0.0001) was noted for the isolates harbouring *bla*_OXA-51_+*bla*_OXA-23_ with *bla*_NDM_ gene alone or co-producing other metallo-β-lactamases (MBLs). When the association of synergism with various phenotypic resistance patterns to other drug classes were compared, no significant association was seen with any profile (**Table 6**).

**Table 5:** Comparison of drug synergy and molecular determinants of CRAB isolates.

Molecular determinants of CRAB	No. of CRAB isolates	Meropenem + Sulbactam		Meropenem + Colistin	
		Synergy n (%)	Indifference n (%)	Synergy n (%)	Indifference n (%)
*bla*_OXA-51_+*bla*_OXA-23_+*bla*_IMP_+*bla*_NDM_+*bla*_VIM_ _*_	20	4 (20)	16 (80)	5 (25)	15 (75)
*bla*_OXA-51_+*bla*_OXA-23_+*bla*_IMP_+*bla*_NDM_ _*_	30	8 (26.66)	22 (73.33)	11 (36.66)	19 (63.33)
*bla*_OXA-51_+*bla*_OXA-23_+*bla*_IMP_+*bla*_VIM_	20	14 (70)	6 (30)	17 (85)	3 (15)
*bla*_OXA-51_+*bla*_OXA-23_+ *bla*_IMP_	10	9 (90)	1 (10)	10 (100)	0
*bla*_OXA-51_+*bla*_OXA-23_+*bla*_NDM_ _*_	10	2 (20)	8 (80)	4 (40)	6 (60)
*bla*_OXA-51_+*bla*_OXA-23_+*bla*_VIM_	5	5 (100)	0	5 (100)	0
*bla*_OXA-51_+*bla*_OXA-23_	5	5 (100)	0	5 (100)	0

*p= <0.0001; Fisher’s exact test applied.

**Table 6:** Comparison of drug synergy and phenotypic non-carbapenem resistance profile of 100 CRAB isolates.

S. no.	Phenotypic resistance profile (n=100)	No. of Isolates n (%)	Meropenem + Sulbactam		Meropenem + Colistin	
			Synergy n (%)	Indifference n (%)	Synergy n (%)	Indifference n (%)
1	AMS^r^/AMK^r^/MIN^r^, TGC^r^	2 (2)	0	2 (100)	0	2 (0)
2	AMS^r^/AMK^r^/MIN^r^	5 (5)	0	5 (100)	1 (20)	4 (80)
3	AMS^r^/AMK^r^/TGC^r^	14 (14)	3 (21.42)	11 (78.57)	2 (14.28)	12 (85.71)
4	AMS ^r^/MIN^r^/TGC^r^	2 (2)	0	2 (100)	0	2 (100)
5	AMK^r^/MIN^r^/TGC^r^	4 (4)	1 (25)	3 (25)	2 (50)	2 (50)
6	AMK^r^/TGC^r^	2 (2)	2 (100)	0	2 (100)	0
7	AMK^r^/MIN^r^	1 (1)	1 (100)	0	1 (100)	0
8	AMK^r^/AMS ^r^	24 (24)	13 (54.16)	11 (78.57)	17 (70.83)	7 (29.16)
9	AMS ^r^/TGC^r^	1 (1)	0	1 (100)	1 ()	0
10	TGC^r^	1 (1)	1 (100)	0	1 (100)	0
11	AMS ^r^	13 (13)	7 (53.84)	6 (46.15)	9 (69.23)	4 (30.76)
12	AMK^r^	27 (27)	19 (70.37)	8 (29.62)	21 (77.77)	6 (22.22)

**^r^** resistance; AMS, ampicillin/sulbactam; AMK, amikacin; MIN, minocycline; TGC, Tigecycline.

### 3.5 Molecular typing

Based on Rep-PCR, 18 different clusters consisting of 2 to 5 isolates with 100% similarity were detected in the 100 CRAB isolates. Besides, 57 singletons were detected with 50-90% similarity as shown in **Supplementary Figure 1**.

## 4 Discussion

The study highlights the extent of antimicrobial resistance in *A. baumannii*, dissemination of carbapenem resistance determinants and more importantly the synergistic effect of drug combinations on molecular determinants of carbapenem resistance. The study is significant as it is the first extensive study on *in-vitro* susceptibility of alternative drugs and their combinations on a large number of CRAB isolates from this part of the globe. The most striking finding is the existence of multiple carbapenemase encoding genes in these isolates with presence of *bla_NDM _* significantly accounting for the loss of synergy in the combination therapies against these isolates.

Both, intrinsic and acquired class D carbapenemases like *bla_OXA-51_* and *bla_OXA-23_*, are the most prevalent enzymes in CRAB worldwide. A recent study on population structure of CRAB circulating in the US hospital systems under the Study Network of *Acinetobacter* as a Carbapenem-Resistant Pathogen (SNAP) study has revealed the predominance of *bla*_OXA-23_ followed by other class D enzymes. In these isolates, MBLs were rare (Iovleva et al., 2022). The situation is in contrast in South and Southeast Asia where MBLs, with comparatively broader spectrum of activity, are quite prevalent (Hsu et al., 2017). This study showed that not only *bla*_NDM_, others like *bla*_IMP_, which was considered rare in CRAB almost 10 years back, has emerged rapidly (Viehman and Nguyen, 2014).

The endemic burden of CRAB has become a major cause of healthcare associated infections (HAIs) in large referral hospitals worldwide. In this study considerable resistance (70% - >90%) against cephalosporins, fluoroquinolones, cotrimoxazoles, piperacillin/tazobactam, carbapenems, amikacin and ampicillin/sulbactam was revealed. Currently the therapeutic options for CRAB might be cefiderocol or colistin in combination with carbapenems or minocycline or tigecycline (Monnheimer et al., 2021). The study showed appreciable *in-vitro* activity of tigecycline (44.39%), minocycline (85.96%), polymyxin B (89.89%) and colistin (97.48%) against CRAB isolates, the use of which could be rationalized for the most effective management of these isolates. In this regard minocycline, as a non-polymyxin based therapeutic agent, has been promising for treatment of CRAB infections. Two large surveillance-based studies on minocycline activity have shown similarly high susceptibility towards *A. baumannii* isolates. However, both these studies were from developed nations where molecular epidemiology of the isolates were different from the present study (Lashinsky et al., 2017). Nevertheless, minocycline was effective in the CRAB isolates with multiple carbapenemase genes. A systematic review of effectiveness of minocycline treatment reported clinical and microbiological success rates of 72.6% and 60.2% respectively. Most of the infections treated were of pneumonia (Fragkou et al., 2019). Susceptibility against tigecycline, another non-polymyxin therapeutic agent, was also tested, as according to clinical practice guidelines by the Infectious Disease Society of America and the American Thoracic Society (ATS-IDSA), the use of tigecycline for the treatment of ventilator associated pneumonia (VAP) in adult patients is recommended (Kalil et al., 2016). Clinical trials to measure the efficacy of tigecycline with comparators are scarce in literature, though one of the largest case series have shown the utility of early initiation of tigecycline in reducing severity of infections due to XDRAb (Lee et al., 2013). However, there has been concern regarding development of resistance with the use of tigecycline as monotherapy. The present study showed more than 50% resistance against tigecycline, though a similar study conducted in an adjacent country (Nepal) showed 100% susceptibility (Joshi et al., 2017). However, smaller sample size in the latter study could be the reason for the difference.

Comparable rates of polymyxin B and colistin resistance ranging from 0%-4% from a multicenter study in European countries has been reported (Wang et al., 2022). Besides, surveillance data from countries of US and Europe have also documented lower rate of polymyxins resistance even in XDR CRAB (Piperaki et al., 2019; Wang et al., 2022). Similarly, in polymyxin based therapies, a recent meta-analysis demonstrated better clinical response as compared to non-polymyxin based therapies (61.7% vs. 39.3%). However, polymyxins being nephrotoxic, showed more adverse events (Lyu et al., 2020).

It should be emphasized that, besides activity, the most important consideration in the above-mentioned antibiotics is the cost. Most of these drugs (minocycline and polymyxins) are not affordable by the people of the developing countries. Access to antibiotics, availability or purchasing power of the population, burden of secondary infection, inadequate healthcare facilities often are the decisive factors for the choice of treatment of CRAB infections (Joshi et al., 2017).

The increasing carbapenem resistance has restricted the antibiotic armamentarium and so combination therapies are frequently being used to increase the antibiotic coverage against MDRAb and XDRAb. The most appropriate combinations suggested against MDRAb and XDRAb in a handful of reports till date is based on testing on a smaller number of isolates than the present study (Laishram et al., 2017). The combinations are of meropenem, imipenem, amikacin or cefepime with sulbactam as it has an intrinsic affinity for penicillin-binding proteins of *A. baumannii*. The other most suggested combination is colistin with carbapenem or colistin-tigecycline (Ayoub Moubareck and Hammoudi Halat, 2020). Based on the hospital setup in this study where meropenem is made available for the treatment free of cost as a part of government supply, synergistic effect for meropenem-sulbactam and meropenem-colistin combinations was studied. The latter showed 57% synergistic effect against the CRAB isolates. Additionally, the colistin combination therapy in comparison with colistin monotherapy has also been found beneficial for reduction in risk of nephrotoxicity (Ayoub Moubareck and Hammoudi Halat, 2020).

All the four Ambler classes have been described in *A. baumannii* and among them the OXA-type carbapenemases followed by MBLs have been reported as dominant mechanism of resistance around the South and Southeast Asian countries (Hsu et al., 2017). Presence of *bla*_OXA-23_ is one of the common causes of resistance conferring the high level of resistance. Usually, MBLs are less frequently detected in developed regions in contrast to the developing regions like India where multiple carabapenemase encoding genes are found in the CRAB isolates without any compensation in fitness (Sharma et al., 2021). Among the genes, *bla*_OXA-23_ is highly endemic and the most common carbapenemase encoding gene found in India followed by *bla*_NDM_ (Vijayakumar et al., 2020). The *bla*_OXA-51_ gene is known to be a native chromosomal oxacillinase and was present in all the study isolates. The widespread burden of *bla*_OXA-23_ (94%) as seen this study indicates the probable relocation of the gene in chromosome or plasmid (Vijayakumar et al., 2022). The prevalence of *bla*_IMP_, gene has already been reported from this study center previously, though infrequent reports have been found from other countries (Alkasaby and Zaki El Sayed, 2017; Banerjee et al., 2018; Fallah et al., 2014). The *bla*_NDM_ genes was also found in a high percentage (59.62%) of the isolates and are known to be widely disseminated around the globe (Fallah et al., 2014; Alkasaby and Zaki El Sayed, 2017; Vijayakumar et al., 2020). The isolates were found negative for class A beta-lactamases and *bla*_OXA-58_ gene, probably because they are the common mechanism of resistance in European or western countries (Vijayakumar et al., 2022). Additionally, it was interesting to note that the predominance of *bla*_OXA-58_ was replaced by *bla*_OXA-23_ since 2009 in the Mediterranean region, probably due to selective advantage of the latter with higher carbapenemase activity (Djahmi et al., 2014). A more recent study has reported isolates producing *bla*_OXA-23_ alone or coproducing *bla*_OXA-23,_ and*bla*_NDM_ mostly belonged to international clone (IC) IC1 and IC2 among which IC2 is highly transmissible (Vijayakumar et al., 2022).

The correlation of molecular mechanism of resistance with synergy rate is an important aspect which has been less studied. The study noted high rate of synergy against both meropenem-sulbactam and meropenem-colistin combinations when there is absence of *bla*_NDM_ gene. The *bla*_NDM_ gene is known to be most concerning gene among the MBLs because the expression of *bla*_NDM_ genes not only helps in production of high-level beta-lactamases but also favours fitness cost for bacterial growth (López et al., 2019; Sharma et al., 2021). While the *bla*_NDM_ gene was first reported from India more than 10 years back, its widespread dissemination is a serious cause of concern (Kumarasamy et al., 2010). Based on this study it can be inferred that in *bla*_NDM_ endemic regions such combinations might not be appropriate strategy for the management of the CRAB isolates.

The study was not without limitations. It was a single center study though a large number of CRAB isolates were included. In addition, the molecular epidemiology of the isolates was representative of the nation at large as per previous reports. Secondly, this was an *in-vitro* study without any data on the course of actual management of the infections with these isolates. Nevertheless, the study clearly reveals the burden of CRAB with more than one carbapenemase encoding genes, role of *bla*_NDM_ in failure of combination therapy and possible therapeutic options against the resistant isolates.

## 5 Conclusion

The study revealed susceptibility of minocycline (85.96%), polymyxin B (89.89%) and colistin (97.48%) against the CRAB isolates with more than one carbapenemase encoding genes from India. Combinations of meropenem-sulbactam and meropenem-colistin showed 47% and 57% synergy respectively. However, presence of *bla*_NDM_ gene in the CRAB isolates was a significant cause of loss of synergy. Therefore, the *bla*_NDM_ endemic regions must review the treatment options against CRAB infections with alternatives like tigecycline, minocycline and polymyxins. Despite being limited to *in-vitro* data, the study involves one of the largest data on synergy testing against CRAB isolates harbouring multiple classes of carbapenemases and their alternative therapeutic options.

## Data availability statement

The original contributions presented in the study are included in the article/**Supplementary Material**. Further inquiries can be directed to the corresponding author.

## Ethics statement

The studies involving human participants were reviewed and approved by Institute Ethical committee, Institute of Medical Sciences, BHU. Written informed consent to participate in this study was provided by the participants’ legal guardian/next of kin. Written informed consent was obtained from the individual(s) for the publication of any potentially identifiable images or data included in this article.

## Author contributions

SS performed the experiment and wrote the manuscript. TB conceptualized, designed the study and revised the manuscript. GY and AK supervised the study. All authors contributed to the article and approved the submitted version.

## Supplementary material

Supplementary Figure 1 Dendrogram by Rep-PCR of 100 CRAB isolates included in drug synergism testing.
