# Supplementary material for: Susceptibility profile of bla OXA-23 and metallo-β-lactamases co-harbouring isolates of carbapenem resistant Acinetobacter baumannii (CRAB) against standard drugs and combinations
Source: Front Cell Infect Microbiol. 2023 Jan 6;12:1068840. doi: 10.3389/fcimb.2022.1068840 (PMC9853021; doi:10.3389/fcimb.2022.1068840)
Supplement: Supplementary file 1 [file Table_1.docx]

**Supplementary Table 1:** Values of MAR index

| **Pattern**  **(n)** | **Resistance pattern phenotype** | **Isolates**  **(n)** | **Total antibiotics**  **(n)** | **MARI** |
| --- | --- | --- | --- | --- |
| 1 | AMS | 10 | 9 | 1.1 |
| 2 | IPM | 1 |  | 0.1 |
| 3 | TGC | 3 |  | 0.3 |
| 4 | IPM+AMK | 4 |  | 0.4 |
| 5 | AMS+AMK | 6 |  | 0.7 |
| 6 | AMS+TGC | 8 |  | 0.9 |
| 7 | TGC+IPM | 1 |  | 0.1 |
| 8 | IMI+MEM+DOR | 14 |  | 1.6 |
| 9 | IMI+AMS+AMK | 9 |  | 1.0 |
| 10 | IMI+MEM+TGC | 1 |  | 0.1 |
| 11 | IMI+AMK+TGC | 5 |  | 0.6 |
| 12 | MEM+DOR+TGC | 1 |  | 0.1 |
| 13 | IPM+MEM+DOR+AMS | 4 |  | 0.4 |
| 14 | IPM+MEM+DOR+AMK | 23 |  | 2.6 |
| 15 | IPM+MEM+DOR+TGC | 13 |  | 1.4 |
| 16 | IPM+MEM+AMS+AMK | 3 |  | 0.3 |
| 17 | IPM+DOR+AMS+AMK | 4 |  | 0.4 |
| 18 | IPM+DOR+AMK+TGC | 1 |  | 0.1 |
| 19 | IPM+AMS+AMK+MIN | 1 |  | 0.1 |
| 20 | IPM+AMS+AMK+TGC | 7 |  | 0.8 |
| 21 | IPM+MEM+DOR+AMS+AMK | 45 |  | 5.0 |
| 22 | IPM+MEM+DOR+AMK+TGC | 43 |  | 4.8 |
| 23 | IPM+MEM+DOR+AMK+POL | 5 |  | 0.6 |
| 24 | IPM+MEM+DOR+AMS+TGC | 3 |  | 0.3 |
| 25 | IPM+MEM+AMS+AMK+TGC | 5 |  | 0.6 |
| 26 | IPM+MEM+AMS+AMK+MIN | 1 |  | 0.1 |
| 27 | IMI+MEM+DOR+AMS+AMK+MIN | 8 |  | 0.9 |
| 28 | IMIP+MEM+DOR+AMS+AMK+TGC | 56 |  | 6.2 |
| 29 | IPM+MEM+DOR+AMK+TGC+POL | 3 |  | 0.3 |
| 30 | IPM+MEM+DOR+AMS+AMK+MIN+POL | 5 |  | 0.6 |
| 31 | IPM+MEM+DOR+AMS+AMK+POL+COL | 2 |  | 0.2 |
| 32 | IPM+MEM+DOR+AMS+AMK+MIN+TGC | 28 |  | 3.1 |
| 33 | IPM+MEM+DOR+AMS+AMK+POL+TGC | 7 |  | 0.8 |
| 34 | IPM+MEM+DOR+AMS+AMK+MIN+POL+TGC | 6 |  | 0.7 |
| 35 | IPM+MEM+DOR+AMS+AMK+POL+COL+TGC | 5 |  | 0.6 |
| 36 | IPM+MEM+DOR+AMS+AMK+MIN+POL+COL+TGC | 2 |  | 0.2 |
|  | Susceptible isolate | 11 |  |  |
|  | Total | 356 |  |  |

IPM= imipenem, MEM= meropenem, DOR= doripenem, AMS= ampicillin/sulbactam, AMK= amikacin, MIN= minocycline, POL= polymyxin B, COL= colistin, TGC= Tigecycline

**Supplementary Table 2:** Summarized data on genetic profiles and synergism

| **CRAB isolate** | **Checkerboard MIC (µg/ml)** | | | | | | | | | **Genetic determinants** | | | | |
| --- | --- | --- | --- | --- | --- | --- | --- | --- | --- | --- | --- | --- | --- | --- |
|  | **Single agent**  **MIC** | | | **MEM+SUL**  **Combination**  **MIC** | | **MEM**  **+**  **SUL**  **FICI** | **MEM+COL**  **Combination**  **MIC** | | **MEM**  **+**  **COL**  **FICI** | ***bla*_OXA-51_** | **bla_OXA-23_** | ***bla*_IMP_** | ***bla*_VIM_** | ***bla*_NDM_** |
|  | MEM | SUL | COL | MEM | SUL |  | MEM | COL |  |  |  |  |  |  |
| 1 | 8 | 16 | 1 | 2 | 1 | 0.31 | 0.5 | 0.25 | 0.31 | P | P | N | N | N |
| 2 | 8 | 32 | 1 | 2 | 8 | 0.5 | 1 | 0.25 | 0.37 | P | P | N | N | N |
| 3 | 8 | 16 | 1 | 2 | 1 | 0.31 | 0.5 | 0.25 | 0.31 | P | P | N | N | N |
| 4 | 128 | 32 | 2 | 16 | 8 | 0.37 | 1 | 0.25 | 0.13 | P | P | N | N | N |
| 5 | 8 | 32 | 1 | 2 | 2 | 0.31 | 0.5 | 0.25 | 0.31 | P | P | N | N | N |
| 6 | 8 | 32 | 2 | 2 | 8 | 0.50 | 0.5 | 0.25 | 0.18 | P | P | P | N | N |
| 7 | 16 | 32 | 1 | 2 | 8 | 0.37 | 0.5 | 0.25 | 0.28 | P | P | P | N | N |
| 8 | 8 | 32 | 2 | 2 | 8 | 0.50 | 0.5 | 0.25 | 0.18 | P | P | P | N | N |
| 9 | 8 | 32 | 2 | 2 | 8 | 0.50 | 0.5 | 0.25 | 0.18 | P | P | P | N | N |
| 10 | 16 | 32 | 2 | 8 | 16 | 1.00 | 0.5 | 0.25 | 0.15 | P | P | P | N | N |
| 11 | 16 | 32 | 1 | 2 | 8 | 0.37 | 0.5 | 0.25 | 0.28 | P | P | P | N | N |
| 12 | 32 | 64 | 1 | 2 | 4 | 0.13 | 0.5 | 0.25 | 0.26 | P | P | P | N | N |
| 13 | 32 | 32 | 1 | 8 | 4 | 0.37 | 0.5 | 0.25 | 0.26 | P | P | P | N | N |
| 14 | 16 | 32 | 2 | 2 | 4 | 0.25 | 0.5 | 0.25 | 0.15 | P | P | P | N | N |
| 15 | 32 | 64 | 1 | 2 | 16 | 0.31 | 0.5 | 0.25 | 0.26 | P | P | P | N | N |
| 16 | 16 | 32 | 1 | 4 | 8 | 0.5 | 0.5 | 0.25 | 0.28 | P | P | N | P | N |
| 17 | 8 | 32 | 1 | 2 | 8 | 0.5 | 0.5 | 0.25 | 0.31 | P | P | N | P | N |
| 18 | 8 | 32 | 1 | 2 | 8 | 0.31 | 0.5 | 0.25 | 0.31 | P | P | N | P | N |
| 19 | 32 | 32 | 2 | 2 | 8 | 0.31 | 1 | 0.25 | 0.15 | P | P | N | P | N |
| 20 | 16 | 32 | 1 | 2 | 8 | 0.37 | 0.5 | 0.25 | 0.28 | P | P | N | P | N |
| 21 | 128 | 32 | 2 | 16 | 8 | 0.37 | 1 | 0.25 | 0.13 | P | P | N | N | P |
| 22 | 128 | 64 | 2 | 32 | 32 | 0.75 | 64 | 1 | 1 | P | P | N | N | P |
| 23 | 128 | 32 | 2 | 32 | 16 | 0.75 | 32 | 1 | 0.75 | P | P | N | N | P |
| 24 | 64 | 128 | 1 | 32 | 128 | 1.5 | 2 | 0.25 | 0.28 | P | P | N | N | P |
| 25 | 64 | 32 | 2 | 16 | 4 | 0.37 | 4 | 0.25 | 0.18 | P | P | N | N | P |
| 26 | 128 | 64 | 2 | 32 | 64 | 1.2 | 16 | 2 | 1.12 | P | P | N | N | P |
| 27 | 32 | 64 | 1 | 8 | 16 | 0.5 | 2 | 0.25 | 0.31 | P | P | N | N | P |
| 28 | 128 | 128 | 2 | 64 | 64 | 1 | 64 | 0.5 | 0.75 | P | P | N | N | P |
| 29 | 256 | 128 | 2 | 128 | 128 | 1.5 | 128 | 1 | 1 | P | P | N | N | P |
| 30 | 128 | 64 | 2 | 32 | 64 | 1.2 | 32 | 1 | 0.75 | P | P | N | N | P |
| 31 | 32 | 64 | 0.5 | 16 | 32 | 1 | 0.5 | 0.25 | 0.26 | P | P | P | P | N |
| 32 | 64 | 64 | 2 | 8 | 16 | 0.37 | 8 | 0.25 | 0.25 | P | P | P | P | N |
| 33 | 64 | 32 | 1 | 16 | 4 | 0.37 | 4 | 0.25 | 0.31 | P | P | P | P | N |
| 34 | 128 | 32 | 2 | 32 | 8 | 0.5 | 16 | 0.25 | 0.25 | P | P | P | P | N |
| 35 | 128 | 64 | 2 | 64 | 64 | 1.5 | 64 | 1 | 1 | P | P | P | P | N |
| 36 | 64 | 32 | 1 | 16 | 2 | 0.31 | 8 | 0.25 | 0.37 | P | P | P | P | N |
| 37 | 8 | 32 | 1 | 2 | 4 | 0.37 | 0.5 | 0.25 | 0.18 | P | P | P | P | N |
| 38 | 64 | 128 | 2 | 32 | 64 | 1 | 4 | 1 | 0.18 | P | P | P | P | N |
| 39 | 64 | 8 | 2 | 16 | 1 | 0.37 | 16 | 0.25 | 0.37 | P | P | P | P | N |
| 40 | 128 | 64 | 0.5 | 64 | 32 | 1 | 32 | 0.5 | 0.5 | P | P | P | P | N |
| 41 | 32 | 64 | 0.5 | 8 | 4 | 0.31 | 0.5 | 0.25 | 0.26 | P | P | P | P | N |
| 42 | 128 | 128 | 1 | 64 | 128 | 1.5 | 16 | 0.25 | 0.37 | P | P | P | P | N |
| 43 | 128 | 32 | 1 | 32 | 8 | 0.5 | 16 | 0.25 | 0.37 | P | P | P | P | N |
| 44 | 32 | 8 | 0.5 | 2 | 2 | 0.31 | 1 | 0.25 | 0.28 | P | P | P | P | N |
| 45 | 64 | 32 | 1 | 8 | 8 | 0.37 | 8 | 0.25 | 0.37 | P | P | P | P | N |
| 46 | 64 | 32 | 0.5 | 16 | 2 | 0.31 | 16 | 0.25 | 0.75 | P | P | P | P | N |
| 47 | 64 | 16 | 1 | 16 | 2 | 0.37 | 16 | 0.25 | 0.5 | P | P | P | P | N |
| 48 | 128 | 16 | 1 | 32 | 2 | 0.37 | 32 | 0.5 | 0.5 | P | P | P | P | N |
| 49 | 128 | 128 | 2 | 128 | 64 | 1.5 | 16 | 2 | 1.12 | P | P | P | P | N |
| 50 | 128 | 64 | 1 | 32 | 16 | 0.5 | 16 | 0.5 | 0.62 | P | P | P | P | N |
| 51 | 256 | 128 | 2 | 128 | 64 | 1 | 128 | 1 | 1 | P | P | P | N | P |
| 52 | 128 | 128 | 2 | 64 | 32 | 0.75 | 8 | 0.25 | 0.18 | P | P | P | N | P |
| 53 | 128 | 128 | 1 | 64 | 128 | 1.5 | 32 | 0.25 | 0.5 | P | P | P | N | P |
| 54 | 128 | 128 | 2 | 64 | 64 | 1 | 64 | 1 | 1 | P | P | P | N | P |
| 55 | 32 | 64 | 1 | 8 | 4 | 0.31 | 1 | 0.25 | 0.28 | P | P | P | N | P |
| 56 | 128 | 128 | 2 | 16 | 64 | 0.62 | 4 | 0.25 | 0.15 | P | P | P | N | P |
| 57 | 256 | 128 | 2 | 64 | 64 | 0.75 | 64 | 0.5 | 0.5 | P | P | P | N | P |
| 58 | 128 | 128 | 1 | 32 | 16 | 0.37 | 32 | 0.5 | 0.75 | P | P | P | N | P |
| 59 | 128 | 128 | 1 | 32 | 64 | 0.75 | 16 | 0.25 | 0.37 | P | P | P | N | P |
| 60 | 256 | 128 | 2 | 64 | 128 | 1.25 | 64 | 1 | 0.75 | P | P | P | N | P |
| 61 | 128 | 128 | 2 | 16 | 32 | 0.37 | 1 | 0.25 | 0.13 | P | P | P | N | P |
| 62 | 128 | 128 | 2 | 64 | 64 | 1 | 16 | 0.25 | 0.25 | P | P | P | N | P |
| 63 | 256 | 64 | 1 | 64 | 64 | 1.25 | 64 | 0.5 | 0.75 | P | P | P | N | P |
| 64 | 128 | 64 | 0.5 | 64 | 16 | 0.75 | 64 | 0.25 | 1 | P | P | P | N | P |
| 65 | 256 | 128 | 2 | 64 | 64 | 0.75 | 64 | 0.5 | 0.5 | P | P | P | N | P |
| 66 | 256 | 128 | 1 | 16 | 32 | 0.31 | 64 | 0.5 | 0.75 | P | P | P | N | P |
| 67 | 128 | 128 | 1 | 64 | 64 | 1 | 64 | 0.5 | 1 | P | P | P | N | P |
| 68 | 128 | 128 | 1 | 16 | 64 | 0.62 | 16 | 1 | 1.12 | P | P | P | N | P |
| 69 | 256 | 128 | 2 | 8 | 64 | 0.53 | 8 | 0.25 | 0.15 | P | P | P | N | P |
| 70 | 128 | 64 | 0.5 | 64 | 64 | 1.5 | 4 | 0.25 | 0.53 | P | P | P | N | P |
| 71 | 128 | 128 | 2 | 64 | 16 | 0.62 | 64 | 0.5 | 0.75 | P | P | P | N | P |
| 72 | 128 | 128 | 1 | 32 | 8 | 0.31 | 64 | 0.5 | 1 | P | P | P | N | P |
| 73 | 256 | 128 | 2 | 32 | 64 | 0.62 | 128 | 0.5 | 0.75 | P | P | P | N | P |
| 74 | 256 | 128 | 2 | 64 | 64 | 0.75 | 128 | 1 | 1 | P | P | P | N | P |
| 75 | 128 | 128 | 2 | 64 | 128 | 1.5 | 64 | 0.25 | 0.62 | P | P | P | N | P |
| 76 | 128 | 64 | 1 | 32 | 4 | 0.31 | 32 | 0.5 | 0.75 | P | P | P | N | P |
| 77 | 32 | 64 | 0.5 | 4 | 16 | 0.37 | 1 | 0.25 | 0.53 | P | P | P | N | P |
| 78 | 256 | 64 | 1 | 64 | 32 | 0.75 | 64 | 0.5 | 0.75 | P | P | P | N | P |
| 79 | 128 | 64 | 1 | 16 | 16 | 0.37 | 4 | 0.25 | 0.28 | P | P | P | N | P |
| 80 | 256 | 32 | 1 | 128 | 2 | 0.56 | 128 | 0.5 | 1 | P | P | P | N | P |
| 81 | 256 | 64 | 0.5 | 128 | 32 | 1 | 32 | 0.5 | 1.12 | P | P | P | P | P |
| 82 | 256 | 128 | 1 | 128 | 128 | 1.5 | 128 | 0.5 | 1 | P | P | P | P | P |
| 83 | 256 | 128 | 1 | 64 | 128 | 1.25 | 64 | 0.5 | 0.75 | P | P | P | P | P |
| 84 | 128 | 128 | 1 | 64 | 64 | 1 | 16 | 0.5 | 0.62 | P | P | P | P | P |
| 85 | 128 | 128 | 2 | 64 | 128 | 1.5 | 32 | 0.25 | 0.37 | P | P | P | P | P |
| 86 | 256 | 128 | 1 | 128 | 64 | 1 | 128 | 0.5 | 1 | P | P | P | P | P |
| 87 | 256 | 64 | 2 | 32 | 32 | 0.62 | 4 | 0.25 | 0.14 | P | P | P | P | P |
| 88 | 256 | 128 | 1 | 128 | 64 | 1 | 128 | 0.5 | 1 | P | P | P | P | P |
| 89 | 256 | 128 | 2 | 128 | 128 | 1.5 | 128 | 0.5 | 0.75 | P | P | P | P | P |
| 90 | 256 | 128 | 2 | 64 | 32 | 0.5 | 32 | 1 | 0.62 | P | P | P | P | P |
| 91 | 128 | 32 | 0.5 | 32 | 2 | 0.31 | 32 | 0.25 | 0.75 | P | P | P | P | P |
| 92 | 256 | 128 | 0.5 | 128 | 64 | 1 | 128 | 0.25 | 1 | P | P | P | P | P |
| 93 | 128 | 128 | 1 | 64 | 128 | 1.5 | 64 | 0.5 | 1 | P | P | P | P | P |
| 94 | 256 | 128 | 0.5 | 64 | 64 | 0.75 | 32 | 0.5 | 1.12 | P | P | P | P | P |
| 95 | 128 | 64 | 2 | 32 | 64 | 1.25 | 64 | 1 | 1 | P | P | P | P | P |
| 96 | 128 | 128 | 1 | 16 | 32 | 0.37 | 2 | 0.25 | 0.26 | P | P | P | P | P |
| 97 | 256 | 32 | 0.5 | 32 | 8 | 0.37 | 4 | 0.25 | 0.51 | P | P | P | P | P |
| 98 | 128 | 64 | 2 | 64 | 32 | 1 | 16 | 0.25 | 0.25 | P | P | P | P | P |
| 99 | 256 | 128 | 1 | 128 | 16 | 0.62 | 32 | 0.25 | 0.37 | P | P | P | P | P |
| 100 | 256 | 128 | 2 | 128 | 32 | 0.75 | 128 | 0.25 | 0.62 | P | P | P | P | P |
