# Supplementary figures and images for: Susceptibility profile of bla OXA-23 and metallo-β-lactamases co-harbouring isolates of carbapenem resistant Acinetobacter baumannii (CRAB) against standard drugs and combinations
Source: Front Cell Infect Microbiol. 2023 Jan 6;12:1068840. doi: 10.3389/fcimb.2022.1068840 (PMC9853021; doi:10.3389/fcimb.2022.1068840)

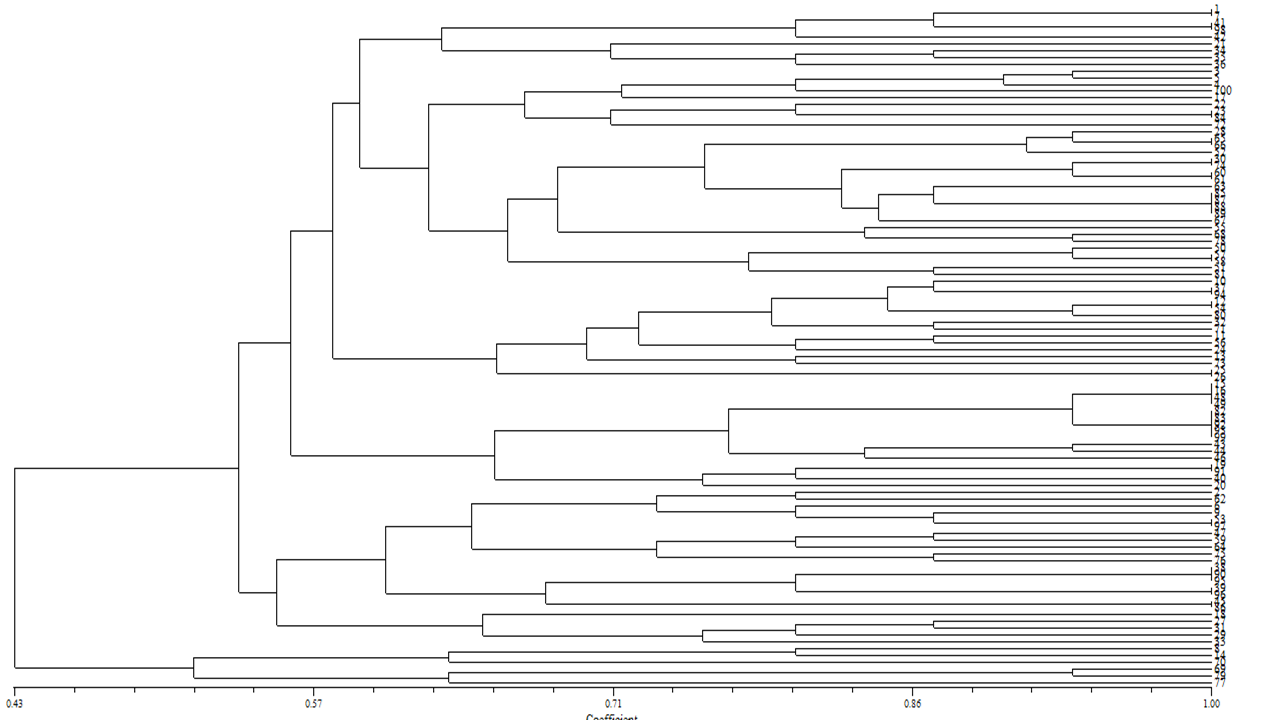

Supplement: Supplementary Figure 1 — Dendrogram by Rep-PCR of 100 CRAB isolates included in drug synergism testing. [file Image_1.tif]
